# Entropy Explains Aging, Genetic Determinism Explains Longevity, and Undefined Terminology Explains Misunderstanding Both

**DOI:** 10.1371/journal.pgen.0030220

**Published:** 2007-12-14

**Authors:** Leonard Hayflick

Communication in the field of biogerontology is a minefield because all of the commonly used terms have no universally accepted definitions. In a series of five annual meetings that I chaired recently in an attempt to define common terms, the dozen or more experts who attended could not agree on the definition of almost all of them, including “aging.” The committee was disbanded and the communications dilemma remains.

“Aging, Bench to Bedside,” the collection of mini reviews published as a series in this journal, is representative of this unsolved problem. Not only does the problem result in communication failures, it also produces erroneous interpretations of research results; illogical allocation of research funds; and misdirected scientific, economic, social, and political policy decisions [1–3]. There is no other field of science in which a similarly bleak situation exists.

As a consequence of the terminology dilemma, I will define for use in this editorial the four aspects of the finitude of life: aging, the determinants of longevity, age-associated diseases, and death. I will not discuss the latter, although even this word defies a universally accepted definition.

## The Aging Process

Age changes can occur in only two fundamental ways: by a purposeful program driven by genes or by random, accidental events.

It is a cornerstone of modern biology that a purposeful genetic program drives all biological processes that occur from the beginning of life to reproductive maturation. However, once reproductive maturation is reached, thought is divided with respect to whether the emerging aging process is a continuation of the genetic program or whether it is the result of the accumulation of random, irreparable losses in molecular fidelity.

The deterministic dream of 19th century physicists was torpedoed in the 20th century with Heisenberg's discovery of the uncertainty principle. In fact, the fundamental laws of physics can only be expressed as probabilities. The most compelling evidence for the belief that biological aging is also a random process is that everything in the universe changes or ages in space-time without being driven by a purposeful program. Although there is no direct evidence that genes drive age changes, their critical role in longevity determination is indisputable.

There is a huge body of knowledge supporting the belief that age changes are characterized by increasing entropy, which results in the random loss of molecular fidelity, and accumulates to slowly overwhelm maintenance systems [1–4].

Both biological systems and inanimate objects incur change over time. Living systems, however, are, among other properties, distinguishable from inanimate objects, because a purposeful genetic program governs the changes that occur from their beginning until reproductive maturation. In inanimate objects, change is not programmed. It is continuous and never ending. Whether the changes that occur in inanimate objects are called age changes or not occurs because of the tendency for humans to view the physical world in anthropomorphic terms.

The common denominator that underlies all modern theories of biological aging is change in molecular structure and, hence, function. These changes are the result of entropic changes, which is now supported by the recent reinterpretation of the Second Law of Thermodynamics, where the belief that it only applies to closed systems has been overturned [5].

Entropy is the tendency for concentrated energy to disperse when unhindered regardless of whether the system is open or closed. The hindrance of entropic change is the relative strength of chemical bonds. The prevention of chemical bond breakage, among other structural changes, is absolutely essential for life. Through evolution, natural selection has favored energy states capable of maintaining fidelity in most molecules until reproductive maturation, after which there is no species survival value for those energy states to be maintained indefinitely.

The dispersal of energy may result in a biologically inactive or malfunctioning molecule. Energy dispersal is never entirely eliminated but it can be circumvented for varying time periods by repair or replacement processes. The internal presence of these repair or replacement processes represents a major difference between living and inanimate forms.

From the standpoint of a physicist, a lowered energy state is not necessarily disorder, because it simply results in the identical molecule with a lowered energy state. The fact that such a molecule might be biologically inactive may not concern the physicist, but it definitely does concern the biologist and, especially, the biogerontologist.

The aging process occurs because the changed energy states of biomolecules renders them inactive or malfunctioning. Identical events also occur before the aging phenotype appears, but repair and replacement processes are capable of maintaining the balance in favor of functioning molecules; otherwise, the species would vanish. After reproductive maturation, this balance slowly shifts to one in which molecules that lose their biologically active energy states are less likely to be replaced or repaired. The diminution of repair and replacement capability is further exacerbated, because the enormously complex biomolecules that compose the repair and replacement systems also suffer the same fate as their substrate biomolecules.

When the escalating loss of molecular fidelity ultimately exceeds repair and turnover capacity, vulnerability to pathology or age-associated diseases increases [1,3,6]. Immortal biological systems cannot exist, if for no other reason than molecular turnover (or dilution) insures that the molecules present at the beginning of a biological lineage are unlikely to be present in that lineage when it reaches Avogadro's Number of about 6 × 10^23^ cells. The only biological property that is long lasting on an evolutionary time scale is the message coded in information-containing molecules, but even that data is subject to mutation or change [7].

Although the loss of molecular fidelity is a random process, there is, nonetheless, a strong element of uniformity, in that errors will occur first in the same families of the most vulnerable molecules in similar cells, organs, or objects. The components of a system in which these molecules are a part then become the weakest link in that system. This accounts for the similarity in the aging phenotype as it progresses within species members.

Similar events occur in aging inanimate objects where, for example, automobiles of a particular make, model, and year of manufacture may have a greater probability of failure in a common weakest link, such as the electrical system. In another car of similar manufacture but different make, year, or model, molecules in the cooling or exhaust system will suffer age changes fastest and become the most probable system to fail first. There is, inevitably, a weakest link with the probability of failing first in a similar component of all complex entities. This “mean time to failure” for a cheap car might be six or seven years, and for newborns today in developed countries their mean time to failure is in the range of 75–85 years.

In humans in developed countries, the weakest links are the cells that compose the vascular system and those in which cancer is most probable. The molecular instability, or aging process, that occurs in these cells is the weakest link that increases vulnerability to these two leading causes of death. This is why knowing how fundamental age changes occur could lead to a better understanding of the etiology of all of the leading causes of death.

The “hypothesis that aging is due in part to mtDNA damage and associated mutations…[because the mitochondrion] generates most cellular ROS” [8] is an excellent example of one of the many possible active causes of the loss of molecular fidelity that characterizes the aging process. Both active and spontaneous entropic processes described above must be balanced by repair and turnover to insure species survival until reproductive success.

Recent studies done using bacteria seem to support the thesis, described above, that “damaged proteins” are the cause of age changes. When a bacterium like Escherichia coli divides by fission, one of the two daughter lineages is “damaged enriched” and the other has “low damage” [9]. The former are “non-culturable or genetically dead” while the latter are “reproductively competent.” In Caulobacter crescentus, replicative senescence has been observed [10], a phenomenon that we first described in normal human cells more than 45 years ago [11]. The phenomenon has also been reported to occur in E. coli and in Saccharomyces cerevisae [12]. The occurrence of replicative senescence in normal cells appears to be a universal biological phenomenon.

## The Determinants of Longevity

The second aspect of the finitude of life is longevity determination—a process that is completely different from aging.

Unlike aging, the genome governs the processes that determine longevity. These are the systems that synthesize molecules and repair or replace them. When the repair or replacement systems are unable to maintain the positive balance that existed prior to reproductive success, a tipping point is reached where the aging phenotype slowly becomes manifest.

Aging must occur in molecules that previously existed with no age changes. It is this prior functional state of molecules and the subsequent efficiency of their maintenance that governs longevity determination.

Unlike the stochastic process that characterizes aging, longevity determination is not a random process. It is governed by the level of physiological reserve reached at the time of reproductive maturation that, through natural selection, was achieved to better guarantee survival to that age. The determination of longevity is incidental to the main goal of the genome, which is to govern events until reproductive maturity occurs. Thus, the genome only indirectly governs longevity.

The variations in excess physiological capacity, repair, and turnover account for the differences found in longevity both within and among species. One might think of longevity determination as the energy state of molecules before they incur age changes, and aging as the state of molecules after energy dissipation results in an irreparable state of functional loss. Longevity determination is a genome-driven anabolic process that addresses the question: “Why do we live as long as we do?” Aging is a chance-driven catabolic process that addresses the question: “Why do things finally go wrong?”

Studies on “dietary restriction” (DR) [13–15] would be better interpreted to have contributed to our understanding of longevity determination than to our understanding of aging. The increase in longevity found by DR does not provide proof that it directly affects the aging process, because longevity is commonly used as the endpoint in these studies, and not age changes.

Increased longevity also could occur if DR eliminated or delayed the appearance of pathology, because biomarkers for aging in most animals are either unknown or not evaluated. Furthermore, because controls are either fed ad lib, or some arbitrary number of calories, it would be just as logical to conclude that overfed animals have a reduced longevity as it would to conclude that DR increases longevity. Indeed, alternating periods of feast and famine is the usual lifestyle for most animals and this is much more likely to mimic the effects of DR. Indeed, DR research might be telling us more about the actual longevity of feral animals absent causes of death attributable to predation, disease, or accidents.

The many studies on gene mutations in C. elegans, drosophila, and other invertebrates [13–15] that have led to the view that genes are involved in aging have not demonstrated that gene manipulation has slowed, stopped, or reversed biomarkers of aging. When all-cause mortality is used as the end point, as is done in experiments with these animals, it cannot be assumed that age changes are being affected. These studies are more accurately interpreted to have an impact on our understanding of longevity determination.

Furthermore, genes that govern the aging process are unnecessary for it to occur. Just as blueprints are vital to construct a complex machine, but contain no information describing a system to cause its aging, the genome is necessary to govern biological development and maintenance, but it contains no instructions to cause the animal to age. Automobiles know how to age without requiring instructions. Both ultimately fail because of changes in molecular fidelity driven by increasing entropy.

In unicellular organisms like yeast, aging has been defined either as the length of time that a yeast cell can survive in a nondividing state, or by the number of daughter cells produced by a mother cell before senescence [13]. In higher animals, chronological time is generally recognized as a poor measure of the rate of aging because of the enormous variations in the aging phenotype among individuals. And, the number of progeny produced before senescence occurs has never been considered to be related to aging. It is more likely that what is being studied are longevity determinants for reasons already given. It has been known for more than a century that longevity determinants in invertebrates are, unlike aging, capable of manipulation.

## Age-Associated Diseases

The third aspect of the finitude of life is age-associated disease. The distinction between the aging process and age-associated disease is not only based on the definition of aging described above, but it is also rooted in several practical observations.

Unlike any disease, age changes:

(1) Occur in every multicellular animal that reaches a fixed size at reproductive maturity.

(2) Cross virtually all species barriers.

(3) Occur in all members of a species only after the age of reproductive maturation.

(4) Occur in all animals removed from the wild and protected by humans even when that species probably has not experienced aging for thousands or even millions of years.

(5) Occur in virtually all animate and inanimate matter.

(6) Have the same universal molecular etiology, that is, thermodynamic instability.

Unlike aging, there is no disease or pathology that shares these six qualities.

The inexorable loss of molecular fidelity that defines aging can either lead to changes that may be nonpathological affronts to vanity, inconveniences, or simply uncomfortable. When the same kind of molecular mischief occurs in the cells of vital organs, leading to an increase in vulnerability to disease or pathology, treatment is required because life may be threatened.

The fundamental aging process is not a disease but it increases vulnerability to disease. Because this critical distinction is generally unappreciated, there is a continuing belief that the resolution of age-associated diseases will advance our understanding of the fundamental aging process [16]. It will not. This is analogous to believing that the successful resolution of childhood pathologies, such as poliomyelitis, Wilms' tumors, and iron deficiency anemia advanced our understanding of childhood development. It did not.

It is often observed that, “The classical evolutionary biological theory of aging tells us that senescence occurs in age-structured populations because of the decline in the force of natural selection with age” [17]. And, a less common belief that, “…the force of natural selection could conceivably increase with age” [17]. These beliefs belie the fact that the forces of natural selection are constant and that large changes usually occur only on an evolutionary time scale. What changes with age is an animal's ability to adapt to the constant forces of natural selection.

The failure to distinguish the fundamental biology of aging (biogerontology) from age-associated pathology (geriatric medicine), and both from longevity determinants, is the most serious impediment to our understanding of the aging process. This failure is exemplified best by realizing that under the rubric “Aging Research,” misled policy makers have appropriated most available funds to research on age-associated diseases. Yet no advance in geriatric medicine will add to our knowledge of the fundamental biology of aging [1–3]. 

## Abbreviations

DR: dietary restriction
